# Male-Specific Transcription Factor Occupancy Alone Does Not Account for Differential Methylation at Imprinted Genes in the *mouse* Germ Cell Lineage

**DOI:** 10.1534/g3.116.033613

**Published:** 2016-09-30

**Authors:** Edward J. Romasko, Nora Engel

**Affiliations:** Fels Institute for Cancer Research and Molecular Biology, Lewis Katz School of Medicine, Temple University, Philadelphia, Pennsylvania 19140

**Keywords:** Genomic imprinting, DNA methylation, transcription factors

## Abstract

Genomic imprinting is an epigenetic mechanism that affects a subset of mammalian genes, resulting in monoallelic expression depending on the parental origin of the alleles. Imprinted regions contain regulatory elements that are methylated in the gametes in a sex-specific manner (differentially methylated regions; DMRs). DMRs are present at nonimprinted loci as well, but whereas most regions are equalized after fertilization, methylation at imprinted regions maintains asymmetry. We tested the hypothesis that paternally unmethylated DMRs are occupied by transcription factors (TFs) present during male gametogenesis. Meta-analysis of *mouse* RNA data to identify DNA-binding proteins expressed in male gametes and motif enrichment analysis of active promoters yielded a list of candidate TFs. We then asked whether imprinted or nonimprinted paternally unmethylated DMRs harbored motifs for these TFs, and found many shared motifs between the two groups. However, DMRs that are methylated in the male germ cells also share motifs with DMRs that remain unmethylated. There are recognition sequences exclusive to the unmethylated DMRs, whether imprinted or not, that correspond with cell-cycle regulators, such as p53. Thus, at least with the current available data, our results indicate a complex scenario in which TF occupancy alone is not likely to play a role in protecting unmethylated DMRs, at least during male gametogenesis. Rather, the epigenetic features of DMRs, regulatory sequences other than DMRs, and the role of DNA-binding proteins capable of endowing sequence specificity to DNA-methylating enzymes are feasible mechanisms and further investigation is needed to answer this question.

One of the outstanding questions in genomic imprinting is how imprints are established. Because all known imprinted loci are associated with at least one CG-rich sequence that is monoallelically methylated (differentially methylated regions; DMRs), DNA methylation is a reasonable surrogate for imprints (Barlow and Bartolomei 2014). The paternally imprinted genes exhibit hypermethylation on the paternal DMR and hypomethylation on the same sequence on the maternal allele, whereas the maternally imprinted genes are associated with hyper- and hypo-methylated DMRs on the maternal and paternal alleles, respectively (Figure 1). Sex-specific marks that result in monoallelic methylation are acquired in the germ cells and are designated gametic DMRs. These include sequences associated with imprinted and nonimprinted genes, but the methylation differences persist after fertilization only for the imprinted DMRs.

**Figure 1:**
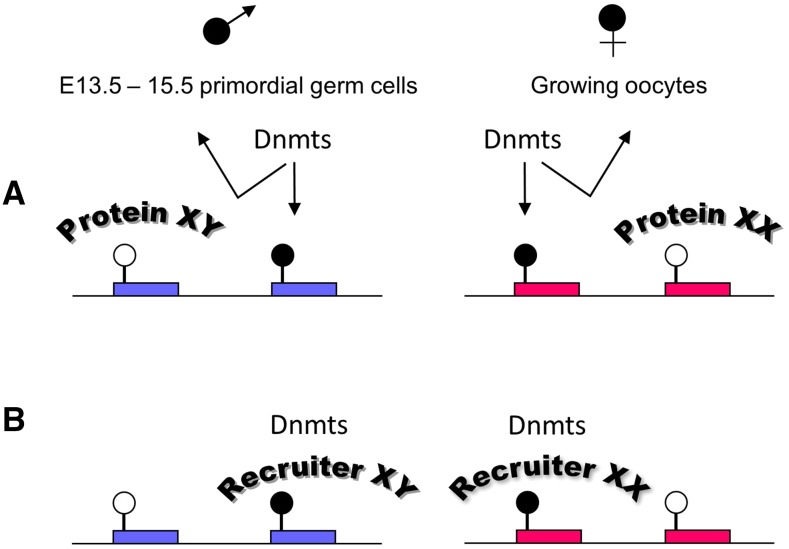
Imprinting establishment hypotheses. (A) DNA-binding proteins expressed in male primordial germ cells or growing oocytes bind different sites, protecting them from methylation. (B) Sequence-specific proteins recognize different sites in males and females and recruit DNA methylation enzymes. Both A and B are valid for both imprinted and nonimprinted DMRs. These hypotheses are not exclusive. Dnmts, DNA methyltransferases.

Many hypotheses have been put forward to explain how DMRs are established (Engel 2015). One possibility is that methylation is acquired in a sequence-specific manner, *i.e.*, that in each germline, distinct elements targeted for methylation are recognized by the methylation machinery. This hypothesis is undermined by the fact that *Dnmt3a* and *Dnmt3l*, the enzymes responsible for establishing methylation imprints, do not have binding specificities (Ooi *et al.* 2009) (Figure 1B). An alternate hypothesis is that at the time of establishment, there is a default, genome-wide methylation event in each germline and specific DNA-binding proteins protect their cognate sequences, some of which are in imprinted regions. The proteins can be transcriptional activators, silencers, pioneer factors—in fact, any type of sequence-specific DNA-binding protein. It is also possible that protection from methylation is due to sex-specific chromatin structures that impede access to the DNA methylation machinery (Figure 1A).

In female germ cells, genome-wide methylation occurs postnatally during oocyte growth. Maternally methylated DMRs are generally intragenic and code for promoters (Barlow and Bartolomei 2014). Abundant data currently supports a model whereby transcriptional activity through DMRs in early-stage oocytes attracts the methylation machinery to those sequences (Chotalia *et al.* 2009; Smallwood *et al.* 2011). The question is: Why do these maternally methylated regions remain unmethylated in the male germline?

In the male, paternally imprinted DMRs acquire DNA methylation during a wave of genome-wide methylation that occurs between 13.5 and 15.5 d postcoitum (dpc), at the prospermatogonia stage (Saitou *et al.* 2012). However, sequences that remain unmethylated, such as promoters for male germ cell–specific genes and the maternally imprinted DMRs, must be protected, *i.e.*, inaccessible to the DNA methylation machinery. Transcription factors (TFs) expressed in the male fetal germ cells are good candidates for blocking their binding sites from DNA methyltransferases. This hypothesis implies that DMRs contain motifs recognized by these TFs, and also that transcriptional activity could be associated with them.

We decided to test the hypothesis that maternally methylated DMRs are protected from methylation on the paternal chromosomes because TFs present in the male germline bind to them (Figure 1A). Our approach is outlined in Figure 2, as follows: (1) we inspected whether there were TFs present in primordial germ cells and prospermatogonia before methylation occurs, using published datasets (Jameson *et al.* 2012; Sakashita *et al.* 2015); (2) since TFs can have low expression levels and might not be represented in the existing datasets, we also identified TF motifs from promoters of genes expressed in primordial germ cells and prospermatogonia; (3) the TFs from steps 1 and 2 were compiled; and (4) we then analyzed a set of paternally hypomethylated gametic DMRs, both imprinted and nonimprinted, to determine if they contain motifs for those TFs and to test whether a distinction could be made between them.

**Figure 2:**
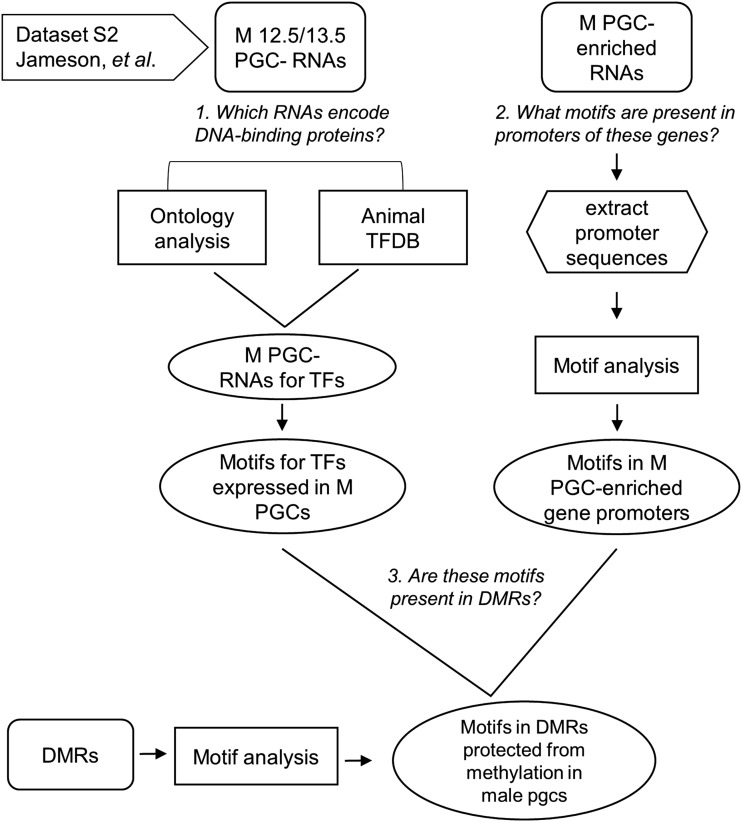
Summary of workflow to identify germ cell-specific TFs and motifs in promoters of genes expressed in male germ cells, to test the hypothesis that DMRs are protected from methylation by occupancy of transcription factors (TFs). Dataset S2 used from Jameson *et al.* (2012). DMR, differentially methylated region; M PGC, male primordial germ cells; TFDB, TF dbase.

Of the 16 domains of imprinted genes, seven of these have been well characterized, five of which contain maternally methylated DMRs, *i.e.*, the imprint was acquired in oogenesis and protected from methylation during spermatogenesis. We restricted our analysis to these five gametic DMRs that have been shown to control imprinting in their cluster by *in vivo* deletion in mutant *mouse* models (Wutz *et al.* 1997; Curley *et al.* 2005; Liu *et al.* 2005; Fitzpatrick *et al.* 2002; Charalambous *et al.* 2010). Nonimprinted gametic DMRs were selected from previous reports (Smallwood *et al.* 2011; Wang *et al.* 2014).

## Materials and Methods

### Retrieval of gene sets

Original gene lists for germ cell– and sex–specifically expressed genes were obtained from Jameson *et al.* (2012). Briefly, the gene lists were generated as follows: gonads were isolated from *Oct4-EGFP* transgenic mice at E11.5, E12.5, and E13.5 (the period when sex determination occurs) from both XX and XY mice. The gonads were FACS-sorted for positive EGFP expression, which indicates their germ cell origin. RNA was purified, analyzed with Affymetrix *Mouse* Genechip Gene 1.0 ST arrays, RMA normalized, and submitted to GEO (Accession number GSE27715). Microarrays were validated by examining expression of particular transcripts previously known to change expression levels in a sex- and/or lineage-specific manner. Multiple pairwise comparisons on the normalized array values generated gene lists that were statistically significantly (*P* < 0.05) enriched or depleted (>1.5-fold), relative to both other lineages and the other sex. For our experiments, gene lists were specifically taken from Dataset S2 in Jameson *et al.* (2012), and these lists were sorted into separate groups based on sex (XX or XY), developmental stage (E12.5 or E13.5; E11.5 was ignored due to its low sequence number), and expression regulation (enriched or depleted).

### Retrieval of promoter sequences

For each gene, (Mouse Genome Informatics MGI) IDs were retrieved from the MGI Microarray Annotation File based on probeset ID (ftp://ftp.informatics.jax.org/pub/reports/Affy_1.0_ST_mgi.rpt) (Eppig *et al*. 2015). Based on MGI IDs, the following data were obtained for each gene, using the MGI Batch Query tool (http://www.informatics.jax.org/batch) with Genome Location as the chosen output: chromosome, strand, start position, end position, and Gene Ontology (GO) terms (based on the December 2011 *Mus musculus* reference genome assembly, GRCm38/mm10). We considered promoter sequences as a 600 bp region containing 500 bp upstream of the transcription start site to 100 bp downstream of the transcription start site (TSS). For genes on the plus (+) strand, the promoter region is evaluated as (Start − 500) to (Start + 100). For genes on the minus (−) strand, the promoter region is evaluated as the reverse complement of (End − 100) to (End + 500). BED files were generated with these data and the UCSC Table Browser data retrieval tool was used to retrieve sequences in FASTA format from the UCSC Genome Browser Database (https://genome.ucsc.edu/) (Karolchik *et al.* 2004). Sequences were obtained both with repetitive sequences masked and unmasked for further analysis by selecting the Mask repeats to *N* option. As a negative control, promoters comprised of random sequences with length of 600 bp were generated. To analyze the role of sequence-specific DNA-binding protein genes, we filtered and counted the genes in each gene set that contained the molecular function accession ID GO:0003700, “transcription factor activity, sequence-specific DNA binding.”

### De novo motif discovery

In order to discover motifs *de novo* within promoters, a local instance of MEME (Multiple Em for Motif Elicitation, version 4.10.0 from http://meme-suite.org/) was used (Bailey and Elkan 1994). As input, MEME was supplied each sample group of DNA sequences in FASTA format. Default parameters were used for all options, with the following exceptions: -dna (for DNA sequences), -mod zoops (assumes zero or one occurrence of the motif per sequence), -nmotifs 3 (for the return of three top scoring motifs), -maxsize 500,000 (for larger character inputs in the case of large number of sequences), and -w X (where X is 6, 8, 10, 12 (or default) to search for motifs of that length).

### Enrichment of known motifs

To identify known motifs significantly enriched within promoters, we used AME (Analysis of Motif Enrichment, version 4.10.0 from http://meme-suite.org/) (Bailey and Elkan 1994). As input, AME was supplied for each sample group of DNA sequences in FASTA format along with those sequences randomly shuffled while maintaining their dinucleotide frequency (generated with the MEME tool fasta-dinucleotide-shuffle). Default parameters were used for all options, with the following exceptions:–bgformat 1 (to set the background source as the MEME motif file), –scoring avg (to score a single sequence for matches to a motif as the average motif score), and –method ranksum (to use the nonparametric Wilcoxon rank-sum association function to test for motif enrichment significance). To search for individual matches of a motif within differentially methylated regions associated with imprinted genes, we used FIMO (Find Individual Motif Occurrences, version 4.10.0 from http://meme.nbcr.net) with the JASPAR Core 2014 vertebrates motif database (205 motifs between 5 and 30 nucleotides in length). We kept only statistically significant motifs with *q*-values < 0.05, where *q*-value is defined as the minimal false discovery rate (FDR) at which a given motif is deemed significant (based on Benjamini and Hochberg 1995). We converted motif IDs to TF name based on ID in the JASPAR database file. The occurrences of each particular motif were counted within each DMR, and these lists were compared to the list of TFs expressed in germ cells.

### Analysis of chromosomal distribution of sex- and stage-specific genes

The Genomic HyperBrowser, a web-based platform based on Galaxy (https://hyperbrowser.uio.no/hb/), was used to perform statistical analyses comparing the chromosomal distribution of genes in the samples (Giardine *et al.* 2005; Blankenberg *et al.* 2010; Goecks *et al.* 2010; Sandve *et al.* 2010). The coordinates within the promoter sequence BED files were first converted from mm10 to mm9 using UCSC LiftOver tool (http://genome.ucsc.edu/cgi-bin/hgLiftOver) for use in Genomic HyperBrowser. To compare the chromosomal frequencies for the sex-specific genes, BED files for the groups were loaded as tracks and hypothesis testing was performed. The null hypothesis was that the expected fraction of points in track one in each chromosome is equal to the expected fraction of points in track two in each chromosome. The alternative hypothesis was that the expected fraction of points of track one in each chromosome was not equal to the expected fraction of points of track two in each chromosome. *P*-values were computed under the null model by preserving the total number of points in both tracks, and randomizing their positions. The test statistic used is the *Z*-statistic based on the observed frequencies, using pooled standard deviation. A collection of FDR-corrected *P*-values (false positives < 10%) per chromosome was computed. Tracks were segments treated as the middle point of every segment. In addition to statistical analyses, the NCBI Genome Decoration Page (http://www.ncbi.nlm.nih.gov/genome/tools/gdp/) was used to visualize chromosomal ideograms annotating where sex- and stage-specific genes mapped to the genome.

### Data availability

Datasets used in this study are publically available and referenced within the article. Data produced by us is available as tables presented within the article and in supplemental tables. Supplemental Material, Table S1 contains the list of TFs expressed in male germ cells, as identified by GO:0003700 and the Animal Transcription Factor Database (www.bioguo.org/AnimalTFDB); Table S2, A and B, contain enriched motifs identified in unmasked and repeat masked promoters, respectively, of genes enriched in E12.5 XX and XY primordial germ cells; Table S2, C and D, contain enriched motifs identified in unmasked and repeat masked promoters, respectively, of genes enriched in E13.5 XX and XY primordial germ cells; Table S3 contains enriched motifs identified in randomly generated control sequences; Table S4 contains the motifs in promoters of genes active in 12.5 and 13.5 dpc primordial germ cells; Table S5 contains the full sequences of paternally unmethylated DMRs associated with analyzed imprinted genes; Table S6 contains the full sequences of paternally unmethylated DMRs associated with analyzed nonimprinted genes; and Table S7 contains the sequences of paternally methylated DMRs associated with imprinted genes analyzed in this study.

## Results

### Identification of RNAs encoding DNA-binding proteins present in male primordial germ cells and prospermatogonia

To tackle the hypothesis that TFs expressed in the male primordial germ cells and/or prospermatogonia protect the unmethylated version of DMRs, we used published microarray data for *mouse* primordial germ cells (Jameson *et al.* 2012). This dataset consists of expression profiles for male and female primordial germ cells and somatic cells at 11.5 and 12.5 dpc, and prospermatogonia, oogonia, and somatic cells at 13.5 dpc. At these stages, methylation imprints have been erased in the germ cells and have not yet been reset (Davis *et al.* 2000). Our workflow is outlined in Figure 2.

To obtain a list of DNA-binding proteins expressed in male primordial germ cells and prospermatogonia, we performed an ontology analysis on the microarray data for 12.5 dpc primordial germ cells and 13.5 dpc prospermatogonia (the 11.5 dpc dataset was not large enough to obtain statistically significant results), using the criteria for “sequence-specific DNA binding TF activity,” obtaining 543 genes (see question 1 in flowchart, Figure 2). We recovered 266 additional TFs by cross-referencing the Animal Transcription Factor Database (http://www.bioguo.org/AnimalTFDB/index.php) with the microarray results (Zhang *et al.* 2015). Table S1 shows the combined list of TFs from both 12.5 and 13.5 dpc, totaling 809. Of those, 20 were enriched in male *vs.* female germ cells and somatic cells.

### Promoter analysis of genes expressed sex-specifically in primordial germ cells, prospermatogonia, and oogonia

Microarray studies could fail to detect key players in transcriptional regulation expressed at low, but functionally significant levels. Thus, we took a second approach to identifying TFs that could block methylation in male germ cells (see question 2 in flowchart, Figure 2). We retrieved promoters of all genes preferentially expressed in primordial germ cells, prospermatogonia, and oogonia compared to somatic cells from Dataset S2 from Jameson *et al.* (2012) to determine the enrichment of TF-binding motifs. The analysis was done with the repeats masked or unmasked, and randomly generated control sequences (Table S2, A–D). The enriched known motif search identified two false positives, motifs for *Tcfap2a* and *Tcfap2c* (Table S3). These were eliminated from the subsequent analyses.

We recovered 28 motifs unique to promoters preferentially active in male primordial germ cells and prospermatogonia, and 117 motifs common to promoters of genes enriched in both male and female primordial germ cells, prospermatogonia, and oogonia relative to somatic cells, for a total of 145 distinct TF motifs (Table S4). A total of 87 of these motifs are for TFs detected in male primordial germ cells and prospermatogonia by microarray, and an additional nine are present in recent RNA-seq data (Sakashita *et al.* 2015). A total of 44 motifs are for TFs not detected in either assay, suggesting that either those TFs have very low expression levels or, alternatively, that they are acting on regulatory sequences other than promoters.

### Identification of motifs in imprinted and nonimprinted gametic DMRs

To test the hypothesis that paternally unmethylated DMRs associated to imprinted and nonimprinted genes are protected during male gametogenesis by TF binding, we looked for motif enrichment in those regions (sequences analyzed in Table S5). Table 1 shows that 33 distinct motifs were identified in five imprinted DMRs that have been shown to control imprinting in their cluster by *in vivo* deletion in mutant *mouse* models, associated with *Nespas*, *Peg3*, *Airn*, *Knq1ot*, and *Grb10* (Wutz *et al.* 1997; Fitzpatrick *et al.* 2002; Curley *et al.* 2005; Liu *et al.* 2005; Charalambous *et al.* 2010). Every DMR shared motifs with at least one other DMR, and four had unique motifs. RNAs for TFs that bind 24 of the 33 motifs (75%) were detected in male primordial germ cells and prospermatogonia, and could be protecting these DMRs from methylation.

**Table 1:** Identification of TF motifs present in DMRs of paternally hypomethylated imprinted genes

DMR	Motif ID	Occurrences	TF Name	TF Expression in Primordial Germ Cells, Spermatogonia, and Oogonia	Motifs Shared with Promoters Active in Male Germ Cells (for TFs Not Detected)	Motifs Unique to the Corresponding Imprinted Gene
*Airn*	MA0024.2	1	E2f1	+	—	—
	MA0073.1	14	Rreb1	+	—	—
	MA0079.3	3	Sp1	+	—	—
	MA0138.2	2	Rest	+	—	—
	MA0163.1	8	Plag1	+	—	—
	MA0469.1	1	E2f3	+	—	—
	MA0471.1	1	E2f6	+	—	—
	MA0516.1	3	Sp2	—	—	—
	MA0528.1	10	Znf263	—	+	—
	MA0599.1	2	Klf5	+	—	—
*Kcnq1ot1*	MA0024.2	1	E2f1	+	—	—
	MA0057.1	1	Mzf1	—	—	+
	MA0079.3	1	Sp1	+	—	—
	MA0146.2	1	Zfx	+	—	—
	MA0162.2	1	Egr1	+	—	—
	MA0163.1	2	Plag1	+	—	—
	MA0516.1	1	Sp2	—	—	—
	MA0591.1	1	Bach1	+	—	—
*Nespas*	MA0024.2	4	E2f1	+	—	—
	MA0035.3	1	Gata1	—	—	+
	MA0036.2	1	Gata2	+	—	+
	MA0079.3	10	Sp1	+	—	—
	MA0092.1	1	Hand1	—	—	+
	MA0112.2	1	Esr1	+	—	—
	MA0146.2	3	Zfx	+	—	—
	MA0162.2	9	Egr1	+	—	—
	MA0163.1	4	Plag1	+	—	—
	MA0469.1	6	E2f3	+	—	—
	MA0470.1	7	E2f4	+	—	—
	MA0472.1	3	Egr2	+	—	—
	MA0499.1	5	Myod1	—	—	+
	MA0500.1	5	Myog	—	—	—
	MA0506.1	3	Nrf1	+	—	—
	MA0516.1	9	Sp2	—	—	—
	MA0521.1	5	Tcf12	+	—	+
	MA0528.1	5	Znf263	—	+	—
	MA0599.1	1	Klf5	+	—	—
*Peg3*	MA0024.2	2	E2f1	+	—	—
	MA0073.1	2	Rreb1	+	—	—
	MA0162.2	1	Egr1	+	—	—
	MA0163.1	1	Plag1	+	—	—
	MA0470.1	2	E2f4	+	—	—
	MA0472.1	1	Egr2	+	—	—
	MA0477.1	1	Fosl1	—	—	+
	MA0489.1	1	Jun	+	—	+
	MA0506.1	1	Nrf1	+	—	—
	MA0528.1	2	Znf263	—	+	—
	MA0591.1	1	Bach1	+	—	—
*Grb10*	MA0525.1	4	Tp63	—	—	+
	MA0139.1	1	Ctcf	+	—	—
	MA0106.2	3	Tp53	—	—	+
	MA0079.3	1	Sp1	+	—	—
	MA0527.1	2	Zbtb33	+	—	+
	MA0146.2	1	Zfx	+	—	—
	MA0599.1	1	Klf5	+	—	—
	MA0048.1	1	Nhlh1	—	+	+
	MA0088.1	1	Znf143	—	—	+
	MA0516.1	1	Sp2	—	—	—
	MA0039.2	1	Klf4	+	—	—

### Identification of motifs in imprinted gametic DMRs that are methylated during male gametogenesis

The ultimate test of our hypothesis was to ask if paternally methylated DMRs lacked motifs for TFs present in male primordial germ cells and prospermatogonia, thus remaining exposed to methylation. There are two gametic DMRs methylated in male primordial germ cells and prospermatogonia, but not in oocytes, that qualify as imprinting control centers, as assayed by loss of imprinted expression in DMR deletion mutant mice, associated with the *H19* and *Dlk1/Gtl2* loci (Table S7, sequences analyzed). We identified 18 distinct motifs present in both DMRs, five of which are shared. Surprisingly, 11 of these motifs are also present either in imprinted or nonimprinted gametic DMRs that remain unmethylated paternally, and 11 of the 18 motifs are binding sites for TFs present at 12.5 dpc male germ cells or 13.5 dpc prospermatogonia (Table 2 and Table 3). Since there are TFs available (or at least represented in the RNA of male primordial germ cells and prospermatogonia) that could protect these DMRs from methylation, the mere presence of TFs in these cells is unable to explain the absence of DNA methylation. Alternatively, the TFs are impeded from binding to their motifs in the *H19* and *Dlk1/Gtl2* DMRs during the methylation wave because of localized chromatin compaction.

**Table 2:** Identification of TF motifs present in gametic DMRs of paternally hypomethylated nonimprinted genes

DMR	Motif ID	Occurrences	TF Name	TF Expression in Primordial Germ Cells, Spermatogonia, and Oogonia	Motifs Shared with Promoters Active in Male Germ Cells (for TFs Not Detected)	Motifs Shared with Imprinted Gametic DMRs
*Shank2*	MA0513.1	1	Smad2	+	—	—
	MA0092.1	1	Hand1	+	—	+
	MA0139.1	1	Ctcf	+	—	+
	MA0024.2	1	E2f1	+	—	+
	MA0145.2	1	Tcfcp2l1	—	—	—
	MA0598.1	1	Ehf	—	+	—
	MA0506.1	1	Nrf1	+	—	+
	MA0048.1	2	Nhlh1	—	+	+
*Ankrd36*	MA0513.1	1	Smad2	+	—	—
	MA0040.1	1	Foxq1	—	—	—
	MA0066.1	1	Pparg	+	—	—
	MA0024.2	1	E2f1	+	—	+
	MA0041.1	1	Foxd3	—	—	—
	MA0163.1	1	Plag1	+	—	+
	MA0500.1	2	Myog	—	—	+
	MA0048.1	1	Nhlh1	—	+	+
	MA0524.1	1	Tfap2c	—	—	—
	MA0146.2	1	Zfx	+	—	+
	MA0521.1	2	Tcf12	+	—	+
	MA0484.1	1	Hnf4g	—	—	—
	MA0030.1	1	Foxf2	—	—	—
	MA0114.2	1	Hnf4a	—	—	—
	MA0525.1	3	Tp63	—	—	+
	MA0106.2	1	Tp53	—	—	+
*Arid1b*	MA0469.1	1	E2f3	+	—	+
	MA0599.1	1	Klf5	+	—	+
	MA0506.1	1	Nrf1	+	—	+
	MA0024.2	1	E2f1	+	—	+
	MA0495.1	1	Maff	+	—	—
	MA0470.1	1	E2f4	+	—	+
	MA0039.2	1	Klf4	+	—	+
	MA0154.2	1	Ebf1	+	—	—
	MA0146.2	2	Zfx	+	—	+
	MA0471.1	1	E2f6	+	—	+
	MA0048.1	2	Nhlh1	—	+	+
	MA0496.1	1	Mafk	—	—	—
	MA0493.1	1	Klf1	—	—	—
	MA0079.3	1	Sp1	+	—	+

Many nonimprinted regions are methylated specifically in oocytes, but not in sperm, thus qualifying as gametic DMRs (Smallwood *et al.* 2011; Wang *et al.* 2014). In contrast to imprinted DMRs, they lose their methylation after fertilization. We selected DMRs associated with three nonimprinted genes, *Shank2*, *Ankrd36*, and *Arid1b* (sequences analyzed in Table S6). We identified 33 motifs in these regions, 17 (51%) of which are shared with motifs present in imprinted DMRs. This suggests that imprinted and nonimprinted DMRs may be protected from methylation by some of the same TFs.

**Table 3:** Identification of TF motifs present in gametic DMRs of paternally methylated imprinted genes

DMR	Motif ID	Occurrences	TF Name	TF Expression in Primordial Germ Cells, Spermatogonia, and Oogonia	Motifs Shared with Imprinted and Nonimprinted Unmethylated Gametic DMRs
*H19*	MA0462.1	1	Batf	—	—
	MA0079.3	1	Sp1	+	+
	MA0599.1	1	Klf5	+	+
	MA0484.1	2	Hnf4a	—	+
	MA0114.2	1	Hnf4g	—	+
	MA0088.1	1	Znf143	—	+
	MA0486.1	1	Hsf1	+	—
	MA0494.1	1	Nr1h3	+	—
	MA0088.3	4	Ctcf	+	+
	MA0144.2	1	Stat2	+	+
*Dlk1/Gtl2*	MA0162.2	1	Egr1	+	+
	MA0073.1	11	Rreb1	+	+
	MA0079.3	3	Sp1	+	+
	MA0142.1	2	Pou5f1	+	—
	MA0088.1	2	Znf143	—	+
	MA0472.1	1	Egr2	+	+
	MA0599.1	4	Klf5	+	+
	MA0484.1	1	Hnf4g	—	+
	MA0519.1	1	Stat5a	—	—
	MA0003.2	2	Tfap2a	—	—
	MA0516.1	3	Sp2	+	+
	MA0114.2	1	Hnf4a	—	+
	MA0144.2	1	Stat3	—	—

We then looked at motifs present in both unmethylated imprinted and nonimprinted DMR motifs that are not present in the *H19* or *Dlk1/Gtl2* DMRs (Table 4). Interestingly, the motifs exclusive to unmethylated DMRs include recognition sites for p53, which is present at the RNA level in male fetal germ cells.

**Table 4:** Motifs common to imprinted and nonimprinted paternally unmethylated gametic DMRs and absent in paternally methylated imprinted genes

TF Name	TF Expression in Primordial Germ Cells, Spermatogonia, and Oogonia
E2f1	+
E2f3	+
E2f4	+
E2f6	+
Hand1	—
Klf4	+
Myog	—
Nhlh1	—
Nrf1	+
Plag1	+
Tcf12	+
Tp53	+
Tp63	—
Zfx	+

## Discussion

Methylation differences between sperm and oocytes are established during gametogenesis, prenatally for males and postnatally for females. In DMRs associated with imprinted genes, these differences are maintained after fertilization, with the methylated alleles resisting the wave of demethylation in preimplantation embryogenesis, and the unmethylated alleles protected from the *de novo* methylation at implantation. How these DMRs differ from gametic nonimprinted DMRs is unclear. One possibility is that the methylated alleles of imprinted DMRs are singled out for protection during genome-wide demethylation in early embryos. There is evidence for the protection of some, but not all, methylated DMRs in imprinted regions after fertilization by the Zfp57 protein (Li *et al.* 2008; Quenneville *et al.* 2011). Also, Zfp57 recognition sites are found at nonimprinted CpG islands (CGIs) that maintain their methylation until implantation (Borgel *et al.* 2010; Smallwood *et al.* 2011). For example, the maternally methylated promoter of *Piwil1* retains its mark until implantation (Kobayashi *et al.* 2012) and, in fact, contains the consensus binding site of Zfp57 (N. Engel, unpublished data). Thus, Zfp57 has a wider protective role in the genome and does not have specificity for methylation at imprinted regions.

In the context of male gametogenesis, *de novo* methylation of DMRs occurs in prospermatogonia starting at ∼15.5 dpc, once they have colonized the gonads, undergone proliferation, and entered mitotic arrest (Saitou *et al.* 2012). Although the genome is highly methylated in mature sperm, most CGIs are unmethylated, suggesting they are sequestered from methylation enzymes in some way (Kobayashi *et al.* 2012). The hypothesis tested here, using currently available data and bioinformatics tools, is that DNA methylation occurs by default wherever CpGs are accessible, but not where protected by the presence of TFs or other factors binding the DMRs. The epigenetic asymmetry between the methylated and unmethylated versions of the DMRs would be the result of a network of TFs specific to each gamete. No distinction would be made between imprinted and nonimprinted unmethylated DMRs at this stage. Rather, the difference between them, *i.e.*, resistance of the imprinted DMRs to methylation after implantation, would be due to specific recognition by protective DNA-binding proteins present in the embryo at that stage.

We recovered several motifs that are common to imprinted and nonimprinted unmethylated DMRs, but are absent in methylated DMRs. Interestingly, they include recognition sites for p53 and p63, both of which are present at the RNA level in male fetal germ cells. Although at present, it is not known whether the p53 protein is expressed and active, male germ cells are arrested in G1, consistent with p53 being active (Sperka *et al.* 2012; Wang *et al.* 2015). Some isoforms of p63 have been found to protect the germline by eliminating oocytes or male germ cells that have suffered DNA damage (Coutandin *et al.* 2016).

Eleven motifs are present exclusively in imprinted paternally unmethylated DMRs. One of them, Bach1, belongs to the basic leucine zipper factor family (bZIP) and also contains a bric-a-brac/poxvirus-zinc finger (BTB/POZ) domain, which facilitates protein-protein interactions. When Bach1 forms a heterodimer with MafK, it functions as a repressor. Both Bach1 and MafK are expressed in male primordial germ cells and spermatogonia, but the Bach1 motif is not present in nonimprinted unmethylated DMRs. Also intriguing is the motif for Zbtb33, which binds to the unmethylated consensus KAISO-binding site TCCTGCNA. Zbtb33 recruits the N-CoR repressor complex to promote histone deacetylation and the formation of repressive chromatin structures.

The observation that weakens the hypothesis is that imprinted gametic DMRs that are paternally methylated also have motifs for TFs present in the male germ cells, but they are not protected from methylation. Analysis of the *H19* and *Dlk1/Gtl2* DMRs predicted 18 TF motifs, some of which were shared with the unmethylated DMRs, and for which 11 TFs are present. The *H19* DMR contains four *CTCF* binding sites and is methylated on the paternal allele (Engel *et al.* 2004; Fedoriw *et al.* 2004). Since CTCF is expressed in male primordial germ cells, it is reasonable to assume that it binds the DMR, and the question arises, why does it not protect from methylation? It had been suggested previously that binding of CTCFL/BORIS in the male germline interferes with CTCF and recruits methylation to *H19* (Loukinov 2002). This is a possible explanation, but no definitive proof for this mechanism has been put forward to date, and the microarray and RNA-seq experiments did not detect expression of CTCFL in the male primordial germ cells or prospermatogonia.

An alternative scenario is that protection of unmethylated DMRs by DNA-binding proteins is a default mechanism and that methylated DMRs are recognized in a sequence-specific or chromatin state–dependent manner and tagged for methylation in combination with factors that result in resistance to demethylation. For example, sequence-specific DNA-binding proteins or noncoding RNAs could guide DNA methyltransferases to the *H19* and *Dlk1/Gtl2* imprinted DMRs, and constitute a complex with additional repressive factors and possibly histone modifiers. Methylated DMRs not associated with imprinted genes would lack these features, rendering them susceptible to post-fertilization demethylation. There is an abundance of zinc-finger proteins and noncoding RNAs with unknown function encoded in the genome and expressed in primordial germ cells and prospermatogonia that could accommodate exclusive recognition of each DMR. These factors could also detect sequences in combination with pre-established chromatin structures unique to the imprinted DMRs, not shared with other elements that are being methylated concurrently across the genome.

Another possibility is that unmethylated DMRs associated with imprinted genes may be engaged in stable physical contacts with other regulatory elements, or isolated in specific topological domains unavailable to methylation enzymes, thus removing them from the genome-wide reprogramming events after fertilization. Modifications to current chromosome conformation assays to analyze low cell numbers are required to further test this proposal.

There are several caveats to our analysis. First, although mRNAs for specific TFs are present, it is possible that the proteins are not, due to post-transcriptional inhibition. Second, motif analysis is continuously being improved due to algorithm development and as more datasets become available, it is possible that revisiting these hypotheses in the future will yield more insight. Third, it is clear that regulatory sequences other than promoters could be involved in protection against methylation, for example, as suggested above, by direct physical contact.

In conclusion, the currently available data does not provide sufficient support for the hypothesis that TFs specifically protect unmethylated DMRs during male gametogenesis without making further assumptions. Even though much progress has been made in identifying the molecular mechanisms of DNA methylation, how it is established selectively for specific CGIs is still an open question.

## Supplementary Material

Supplemental Material

## References

[bib1] Bailey T. L. Elkan C., 1994 Fitting a mixture model by expectation maximization to discover motifs in biopolymers. Proc. Int. Conf. Intell. Syst. Mol. Biol. 2: 28–36. 7584402

[bib2] Barlow D. P. Bartolomei M. S., 2014 Genomic imprinting in mammals. Cold Spring Harb. Perspect. Biol. 6: a018382. 24492710 10.1101/cshperspect.a018382 PMC3941233

[bib33] Benjamini Y. Hochberg Y., 1995 Controlling the False Discovery Rate: a practical and powerful approach to multiple testing. J. Royal Stat. Soc. Series. B, 57: 289–300.

[bib3] Blankenberg D. Von Kuster G. Coraor N. Ananda G. Lazarus R., 2010 Galaxy: a web-based genome analysis tool for experimentalists. Curr Protoc Mol Biol Chapter 19, Unit 19: 10.1–21. 10.1002/0471142727.mb1910s89 PMC4264107 20069535

[bib4] Borgel J. Guibert S. Li Y. Chiba H. Schubeler D., 2010 Targets and dynamics of promoter DNA methylation during early *mouse* development. Nat. Genet. 42: 1093–1100. 21057502 10.1038/ng.708

[bib5] Charalambous M. Cowley M. Geoghegan F. Smith F. M. Radford E. J., 2010 Maternally-inherited Grb10 reduces placental size and efficiency. Dev. Biol. 337: 1–8. 19833122 10.1016/j.ydbio.2009.10.011

[bib6] Chotalia M. Smallwood S. A. Ruf N. Dawson C. Lucifero D., 2009 Transcription is required for establishment of germline methylation marks at imprinted genes. Genes Dev. 23: 105–117. 19136628 10.1101/gad.495809 PMC2632167

[bib7] Coutandin D. Osterburg C. Srivastav R. K. Sumyk M. Kehrloesser S., 2016 Quality control in oocytes by p63 is based on a spring-loaded activation mechanism on the molecular and cellular level. eLife 5: e13909. 27021569 10.7554/eLife.13909 PMC4876613

[bib8] Curley J. P. Pinnock S. B. Dickson S. L. Thresher R. Miyoshi N., 2005 Increased body fat in mice with a targeted mutation of the paternally expressed imprinted gene Peg3. FASEB J. 19: 1302–1304. 15928196 10.1096/fj.04-3216fje

[bib9] Davis T. L. Yang G. J. McCarrey J. R. Bartolomei M. S., 2000 The H19 methylation imprint is erased and re-established differentially on the parental alleles during male germ cell development. Hum. Mol. Genet. 9: 2885–2894. 11092765 10.1093/hmg/9.19.2885

[bib10] Engel N., 2015 Genomic imprinting in mammals: memories of generations past, in *Epigenetic Gene Expression and Regulation Chap 3*, edited by Litt M. D. Blakely C. A. Elsevier Press, New York, NY.

[bib11] Engel N. West A. G. Felsenfeld G. Bartolomei M. S., 2004 Antagonism between DNA hypermethylation and enhancer-blocking activity at the H19 DMD is uncovered by CpG mutations. Nat. Genet. 36: 883–888. 15273688 10.1038/ng1399

[bib34] Eppig J. T. Blake J. A. Bult C. J. Kadin J. A. Richardson J. E., 2015 The Mouse Genome Database Group. 2015 The Mouse Genome Database (MGD): facilitating mouse as a model for human biology and disease. Nucleic Acids Res. Jan 28;43(Database issue):D726-36.

[bib12] Fedoriw A. M. Engel N. I. Bartolomei M. S., 2004 Genomic imprinting: antagonistic mechanisms in the germ line and early embryo. Cold Spring Harb. Symp. Quant. Biol. 69: 39–45. 16117631 10.1101/sqb.2004.69.39

[bib13] Fitzpatrick G. V. Soloway P. D. Higgins M. J., 2002 Regional loss of imprinting and growth deficiency in mice with a targeted deletion of KvDMR1. Nat. Genet. 32: 426–431. 12410230 10.1038/ng988

[bib14] Giardine B. Riemer C. Hardison R. C. Burhans R. Elnitski L., 2005 Galaxy: a platform for interactive large-scale genome analysis. Genome Res. 15: 1451–1455. 16169926 10.1101/gr.4086505 PMC1240089

[bib15] Goecks J. Nekrutenko A. Taylor J. Team Galaxy, 2010 Galaxy: a comprehensive approach for supporting accessible, reproducible, and transparent computational research in the life sciences. Genome Biol. 11: R86. 20738864 10.1186/gb-2010-11-8-r86 PMC2945788

[bib16] Jameson S. A. Natarajan A. Cool J. DeFalco T. Maatouk D. M., 2012 Temporal transcriptional profiling of somatic and germ cells reveals biased lineage priming of sexual fate in the fetal *mouse* gonad. PLoS Genet. 8: e1002575. 22438826 10.1371/journal.pgen.1002575 PMC3305395

[bib17] Karolchik D. Hinrichs A. S. Furey T. S. Roskin K. M. Sugnet C. W., 2004 The UCSC table browser data retrieval tool. Nucleic Acids Res. 32: D493–D496. 14681465 10.1093/nar/gkh103 PMC308837

[bib18] Kobayashi H. Sakurai T. Imai M. Takahashi N. Fukuda A., 2012 Contribution of intragenic DNA methylation in *mouse* gametic DNA methylomes to establish oocyte-specific heritable marks. PLoS Genet. 8: e1002440. 22242016 10.1371/journal.pgen.1002440 PMC3252278

[bib19] Li X. Ito M. Zhou F. Youngson N. Zuo X., 2008 A maternal-zygotic effect gene, Zfp57, maintains both maternal and paternal imprints. Dev. Cell 15: 547–557. 18854139 10.1016/j.devcel.2008.08.014 PMC2593089

[bib20] Liu J. Chen M. Deng C. Bourc’his D. Nealon J. G., 2005 Identification of the control region for tissue-specific imprinting of the stimulatory G protein alpha-subunit. Proc. Natl. Acad. Sci. USA 102: 5513–5518. 15811946 10.1073/pnas.0408262102 PMC556240

[bib21] Loukinov D. I. Pugacheva E. Vatolin S. Pack S. D. Moon H., 2002 BORIS, a novel male germ-line-specific protein associated with epigenetic reprogramming events, shares the same 11-zinc-finger domain with CTCF, the insulator protein involved in reading imprinting marks in the soma. Proc. Natl. Acad. Sci. USA 99: 6806–6811. 12011441 10.1073/pnas.092123699 PMC124484

[bib22] Ooi S. K. O’Donnell A. H. Bestor T. H., 2009 Mammalian cytosine methylation at a glance. J. Cell Sci. 122: 2787–2791. 19657014 10.1242/jcs.015123 PMC2724605

[bib23] Quenneville S. Verde G. Corsinotti A. Kapopoulou A. Jakobsson J., 2011 In embryonic stem cells, ZFP57/KAP1 recognize a methylated hexanucleotide to affect chromatin and DNA methylation of imprinting control regions. Mol. Cell 44: 361–372. 22055183 10.1016/j.molcel.2011.08.032 PMC3210328

[bib24] Saitou M. Kagiwada S. Kurimoto K., 2012 Epigenetic reprogramming in *mouse* pre-implantation development and primordial germ cells. Development 139: 15–31. 22147951 10.1242/dev.050849

[bib25] Sakashita A. Kawabata Y. Jincho Y. Tajima S. Kumamoto S., 2015 Sex specification and heterogeneity of primordial germ cells in mice. PLoS One 10: e0144836. 26700643 10.1371/journal.pone.0144836 PMC4689518

[bib26] Sandve G. K. Gundersen S. Rydbeck H. Glad I. K. Holden L., 2010 The genomic hyperbrowser: inferential genomics at the sequence level. Genome Biol. 11: R121. 21182759 10.1186/gb-2010-11-12-r121 PMC3046481

[bib27] Smallwood S. A. Tomizawa S. Krueger F. Ruf N. Carli N., 2011 Dynamic CpG island methylation landscape in oocytes and preimplantation embryos. Nat. Genet. 43: 811–814. 21706000 10.1038/ng.864 PMC3146050

[bib28] Sperka T. Wang J. Rudolph K. L., 2012 DNA damage checkpoints in stem cells, ageing and cancer. Nat. Rev. Mol. Cell Biol. 13: 579–590. 22914294 10.1038/nrm3420

[bib29] Wang L. Zhang J. Duan J. Gao X. Zhu W., 2014 Programming and inheritance of parental DNA methylomes in mammals. Cell 157: 979–991. 24813617 10.1016/j.cell.2014.04.017 PMC4096154

[bib30] Wang X. Simpson E. R. Brown K. A., 2015 p53: protection against tumor growth beyond effects on cell cycle and apoptosis. Cancer Res. 75: 5001–5007. 26573797 10.1158/0008-5472.CAN-15-0563

[bib31] Wutz A. Smrzka O. W. Schweifer N. Schellander K. Wagner E. F., 1997 Imprinted expression of the *Igf2r* gene depends on an intronic CpG island. Nature 389: 745–749. 9338788 10.1038/39631

[bib32] Zhang H. M. Liu T. Liu C. J. Song S. Zhang X., 2015 AnimalTFDB 2.0: a resource for expression, prediction and functional study of animal transcription factors. Nucleic Acids Res. 43: D76–D81. 25262351 10.1093/nar/gku887 PMC4384004

