# Supplementary material for: Male-Specific Transcription Factor Occupancy Alone Does Not Account for Differential Methylation at Imprinted Genes in the mouse Germ Cell Lineage
Source: G3 (Bethesda). 2016 Sep 30;6(12):3975–83. doi: 10.1534/g3.116.033613 (PMC5144967; doi:10.1534/g3.116.033613)
Supplement: Supplemental Material [file supp_6_12_3975__index.html]

Male-Specific Transcription Factor Occupancy Alone Does Not Account for Differential Methylation at Imprinted Genes in the mouse Germ Cell Lineage — Supplemental Material 

# Male-Specific Transcription Factor Occupancy Alone Does Not Account for Differential Methylation at Imprinted Genes in the *mouse* Germ Cell Lineage

## Supplemental Material for Romasko and Engel, 2016

**Files in this Data Supplement:**

- Table S1 - Transcription factors expressed in male germ cells as identified by GO:0003700 and the Animal Transcription factor database (www.bioguo.org/AnimalTFDB). (.xlsx, 20 KB)
- Table S2 - Enriched motifs identified in repeat masked promoters of genes enriched in E13.5 XX and XY primordial germ cells. (.xlsx, 52 KB)
- Table S3 - Enriched motifs identified in randomly generated control sequences. (.xlsx, 14 KB)
- Table S4 - Motifs in promoters of genes from 12.5 and 13.5 dpc primordial germ cells. (.xlsx, 16 KB)
- Table S5 - Sequences of paternally unmethylated DMRs. (.xlsx, 15 KB)
- Table S6 - Sequences of paternally unmethylated DMRs. (.xlsx, 13 KB)
- Table S7 - Sequences of paternally methylated DMRs associated with imprinted genes analyzed in this study (locations are in mm9). (.xlsx, 15 KB)
