# Supplementary material for: Male-Specific Transcription Factor Occupancy Alone Does Not Account for Differential Methylation at Imprinted Genes in the mouse Germ Cell Lineage
Source: G3 (Bethesda). 2016 Sep 30;6(12):3975–83. doi: 10.1534/g3.116.033613 (PMC5144967; doi:10.1534/g3.116.033613)
Supplement: Supplemental Material [file supp_g3.116.033613_TableS4.docx]

Table S4. Motifs in promoters of genes from 12.5 and 13.5 dpc primordial germ cells. Includes those unique to XY (in blue) and common to both XX and XY. (.xlsx, 16 KB)

<http://www.g3journal.org/lookup/suppl/doi:10.1534/g3.116.033613/-/DC1/TableS4.xlsx>
