# Supplementary material for: Male-Specific Transcription Factor Occupancy Alone Does Not Account for Differential Methylation at Imprinted Genes in the mouse Germ Cell Lineage
Source: G3 (Bethesda). 2016 Sep 30;6(12):3975–83. doi: 10.1534/g3.116.033613 (PMC5144967; doi:10.1534/g3.116.033613)
Supplement: Supplemental Material [file supp_g3.116.033613_TableS5.docx]

Table S5. Sequences of paternally unmethylated DMRs. (.xlsx, 15 KB)

<http://www.g3journal.org/lookup/suppl/doi:10.1534/g3.116.033613/-/DC1/TableS5.xlsx>
